# Non-adaptive strategy selection in adults with high mathematical anxiety

**DOI:** 10.1038/s41598-018-27763-w

**Published:** 2018-07-16

**Authors:** Sarit Ashkenazi, Deema Najjar

**Affiliations:** Learning Disabilities, the Seymour Fox School of Education, the Hebrew University of Jerusalem, Mount Scopus, Jerusalem, 91905 Israel

## Abstract

Participants with mathematical anxiety (MA) tend to show particular difficulty in mathematical operations with high working memory (WM) demands compared to operations with lower WM demands. Accordingly, we examined strategy selection to test the cognitive mechanism underlying the observed weakness of high MA participants in mathematical operations with high WM demands. We compared two groups of college students with high or low MA, in the solution of simple non-carry addition problems (e.g., 54 + 63) and complex carryover addition problems (e.g., 59 + 63). The results indicated that high MA participants showed particular difficulty in the harder carry condition. Testing the strategy selection mechanism among high MA participants, we found in the carry condition 1) they used the common strategy less often compared to low MA participants and 2) employed unusual strategies more often compared to low MA participants. Therefore, high MA participants were less efficient in their strategy selection, which may be due to weaker spatial representations, numerical difficulties, or less experience solving complex problems. These primitive representations are not adaptive, and can negatively impact performance in math tasks with high WM demands.

## Introduction

Mathematical anxiety (MA) is a feeling of tension and anxiety that interferes with the manipulation of numbers and the solution of mathematical problems in a wide variety of everyday life and academic situations^1^. There is an ongoing debate in relation to the origin of MA, and the situational influences of MA in the case of high anxiety^2–4^. Some studies found that MA originates from a weakness in basic numerical processing that is mediated by impaired spatial abilities^5,6^. While others have found that MA influences working memory (WM), and math performance^2–4^. According to this line of studies, poor math performance among people with MA is a result of limited WM resources, which are required for the solution of math problems, due to anxiety induced verbal ruminations^2–4^. The affective drop in performance is observed when math is performed under timed, high-stakes conditions^2,7^. Hence, when people with MA perform arithmetic tasks that place high demands on WM their performance is worse compared to tasks with lower WM demands. Similar to the affective drop in performance theory, the attentional control theory proposes that MA, similar to anxiety disorders, affects central executive WM, thus reducing inhibition and shifting abilities^8^.

Contrary to the limited WM resources theory regarding MA^2,7,8^, studies have found that participants with high MA (HMA) had lower performance compared to participants with low MA (LMA) in basic numerical tasks (such as symbolic and non-symbolic comparison tasks) related to number sense, which have minimal WM demands^9–11^. Number sense is an understanding of approximate quantities and the relation between quantities, grounded in a spatial representation^12–14^. Some studies found that a weakness in spatial processing was the origin of the number sense deficit in HMA participants. Ferguson *et al*.^15^ found a strong negative correlation between spatial abilities and MA. The same study also found that participants with HMA reported a worse sense of direction and more spatial anxiety than their LMA peers^15^. Moreover, participants with HMA had lower mental rotation abilities than LMA participants^15^. Last, participants with HMA had a stronger mental representation that binds numbers and space (abnormal numerical distance effect and a spatial-numerical association of response codes effect)^5^. Weakness in spatial abilities can result in abnormal basic numerical representation or weakness in number sense, and thus may contribute to MA.

One way to understand the origin of MA, and the situational influences of MA in the case of high anxiety, is to test strategy selection (i.e., choice of appropriate strategies in relation to task characteristics) and strategy execution (i.e., performance of the cognitive processes involved in each strategy) during the solution of math problems (for an explanation of the common strategies employed during complex arithmetic problem see our explanation of the task below). From early childhood, children constantly learn and acquire new strategies. The new strategies result from conceptual understanding of the requisites of appropriate strategies for a specific problem^16^. Flexibility in problem solving will result from knowledge of (a) multiple strategies and (b) the relative efficiency of these strategies^17,18^. Research on mathematical strategy selection and strategy execution has differentiated between groups based on arithmetical capabilities, WM capacity, age and counting knowledge^19–21^. For instance, children with mathematical learning disabilities used strategies that were developmentally below age level during the solution of simple arithmetic problems (e.g., 7 + 8), such as counting all (count both addends) and using fingers as counting aids. Moreover, their strategy selection was poor and they continued to use the same strategy regardless of problem type^22^.

Among adults, mathematical experts tend to use a larger variety of strategies and more advanced strategies compared to non-experts^23^. Hodzik and Lemaire^24^ found that young and older adults differed in how many strategies they used, as well as in inhibition and shifting capabilities during complex arithmetical problem solution, which mediated age-related differences in strategy repertoire and strategy selection (for a similar finding see^25^). Using a similar methodology to Hodzik and Lemaire^24^, with the addition of WM manipulation, we found that strategy selection was modulated by WM demand, number sense ability and central executive abilities^26^.

Very few studies directly examined strategy selection among HMA participants^27,28^. One study found that children with HMA used retrieval less often and less frequently than their low MA peers^27^. Hence, similar to participants with learning disabilities, individuals with HMA tended to employ backup procedural strategies rather than direct retrieval^19^, which is less efficient. In a study with children with MA, Ramirez *et al*.^29^ grouped the HMA participants based on WM abilities. They found that participants with high WM abilities tended to rely on advanced strategies (such as direct retrieval or decomposition), while participants with low WM abilities relied on strategies with minimal WM demand (such as counting all). The use of advanced memory based strategies partially mediated the relationship between MA and math achievement: advanced strategies are prone to suffer from the fact that WM capacity is co-opted by MA (for similar results in adults see^30–32^). Hence, children with MA and high WM capabilities have poorer performance in mathematics tasks with high WM demands because they cannot access strategies they would normally rely on.

Si, *et al*.^28^ tested the effect of MA on strategy choice in computational estimation and mental arithmetic tasks, and examined age-related differences. The results indicated that MA had a greater effect on computational estimation than on mental arithmetic. Moreover, MA had a greater affect on 6th grade students compared to 4th graders and adults.

The goal of the present study was to explore strategy selection and repertoire in participants with HMA, in order to understand the origin of MA and the influence of MA on the solution of math operations. MA was expected to interfere with the solution of operations that involved high WM load but not low WM load. Accordingly, we manipulated WM resources by comparing operations with no carryover (low WM load) to operations with carryover (high WM load). Ashcraft and Kirk^3^ found that participants with HMA had particular difficulty with carryover operations. Hence, we expected to find larger group differences in solution efficiency and strategy selection for carryover problems compared to non-carryover problems. It was reported that both numerical and executive function abilities affect strategy selection and solution efficiency^26^. Hence, we explored the respective roles of non-symbolic comparison and central executive abilities as possible explanatory factors for individual differences in efficiency of solution and strategy selection. We believe that the effects of non-symbolic comparison and central executive abilities will be mediated by the MA group (we found in a previous study that these abilities affect strategy selection in the normative population^26^).

There is an ongoing debate in relation to the origin of MA, and the situational influences of MA in the case of high anxiety. One approach suggests that MA is based on an impairment in quantity representation that is mediated by spatial weakness, which resulted in poorer math performance among individuals with HMA^5,10^. While others posit that anxiety itself is the origin of reduced math performance among individuals with HMA; anxiety reduces executive functions abilities (attentional control capacity)^2,3,8^. Hence, seemingly contradictory predictions can be hypothesized based on these theories. If the deficit in numerical processing is the heart of MA then we should expect to find that the HMA participants will select inappropriate, unusual strategies^5^. However, if MA reduces cognitive control^2,3,8^, then participants with HMA should use a smaller number of strategies and use them less effectively compared to participants with LMA^24^ (but see Dowker^23^ for contradictory predictions related to larger strategic variability with high proportions of non-sophisticated strategies among math novices compared to math middle range of expertise).

## Results

The data analysis included several stages. First, we compared the HMA and LMA participants performance in the non-symbolic comparison task and an executive function task. Then, we analyzed strategy selection in each group. Afterwards, we examined solution efficiency for complex addition problems by group and problem type, and tested proportion of use of the ten common strategies by problem type and group. Finally, to test strategic variation we analyzed the number of strategies used by problem type and group.

- **Group comparison of general abilities**
  The two groups had comparable performance in non-symbolic comparison and the Tower of Hanoi (see Table 1).

**Table 1:** Executive functions, quantity discrimination and complex addition performance by group.

	HMA		LMA		p
	*Mean*	*S.D*.	*Mean*	*S.D*.	
Tower of Hanoi	2.90	1.98	2.30	2.01	0.24
Weber fraction	0.36	0.23	0.37	0.47	0.86
Non-symbolic comparison accuracy	74.87	7.67	76.86	10.33	0.46
Non-symbolic comparison RT	914.59	327.54	863.08	318.56	0.58
Accuracy addition non-carry	0.81	0.11	0.84	0.12	0.35
Accuracy addition carry	0.73	0.14	0.78	0.16	0.19
RTs addition non-carry	6,982.25	2,751.23	5,050.12	2,126.05	0.001
RTs addition carry	10,850.56	4,402.35	6,878.74	3,076.36	0.001
- **Strategy selection**

Global analysis of the favorite strategy (the strategy used with the greatest frequency) (see Table 2 for all of the strategies) showed that columnar retrieval was used in more than half (56%) of all trials. Analysis of the favorite strategy in each group showed that columnar retrieval was used the most often in HMA (51%) and LMA (62%), followed by vertical imagery in HMA (16%) and rounding the two operands down (10%) in LMA. Finally, the HMA group used rounding the second operand up and rounding the two operands down (8% for both) while the LMA group used vertical imagery and rounding the second operand (6% for each strategy).

**Table 2:** Average percentage use of each strategy in each group.

Strategy	1	2	3	4	5	6	7	8	9	10	11	12	13
HMA	0.5	1.0	7.8	50.6	5.0	7.9	0.1	0.3	0.2	4.1	16.0	2.8	3.8
LMA	0.5	1.3	9.5	61.9	4.8	6.1	0.4	3.0	0.0	0.1	6.1	0.5	5.9

1 = rounding the first operand down 2 = rounding the second operand down 3 = rounding both operand down 4 = columnar retrieval 5 = rounding the first operand up 6 = rounding the second operand up 7 = rounding the two operands up 8 = unit addition 9 = retrieval 10 = others 11 = vertical imagery 12 = decomposition of the units 13 = decomposition of the decade.

### Accuracy rate

**Figure 1:**
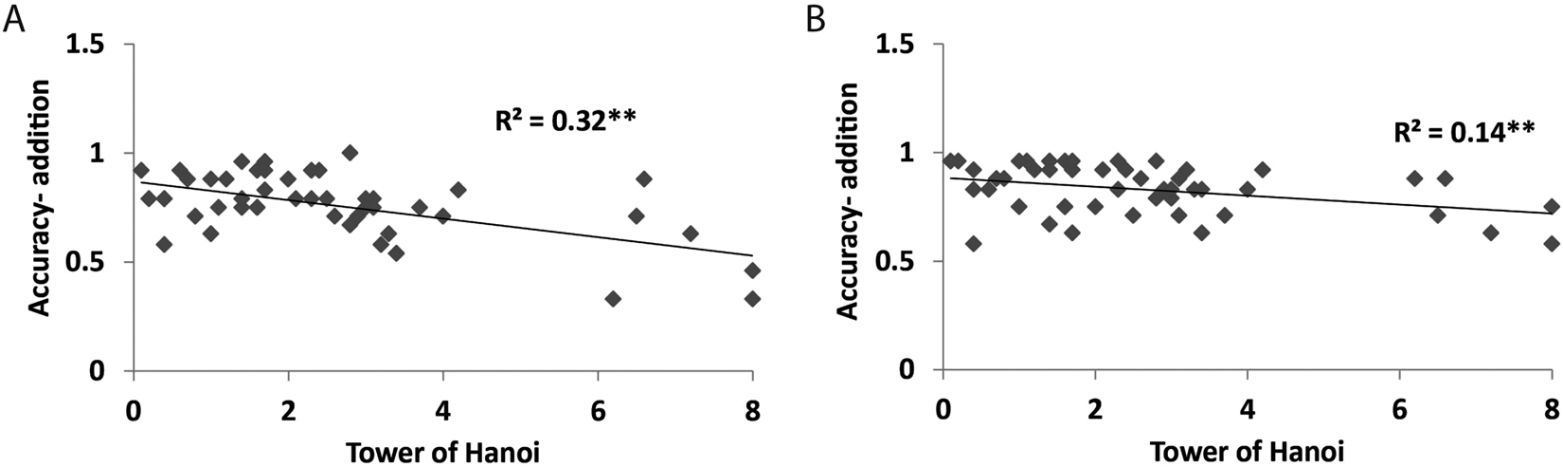
High executive function ability associated with high accuracy in math problem solution. The figure presents the correlation between tower of Hanoi scores and accuracy rates in math operations. Better executive function ability was associated with high accuracy. The correlation was stronger in the carry condition (**A**) compared to the no carry condition (**B**).

A two-way analysis of covariance (ANCOVA) was performed on the accuracy rates with problem type (carry or no-carry) as the within subject factor and group (HMA or LMA) as the between subject factor. Tower of Hanoi and accuracy in non-symbolic comparison were the covariates. The only significant effect was Tower of Hanoi, *F*(1, 44) = 18.09, partial *η*^2^ = 0.29, *p* < 0.01. The interaction between Tower of Hanoi and problem type also reached significance, *F*(1, 44) = 4.36, partial *η*^2^ = 0.09, *p* < 0.05. Better performance in the Tower of Hanoi was associated with higher accuracy both in carry and no carry problems *r*(48) = −0.56, *p* < 0.001 and *r*(48) = −0.37, *p* < 0.01 (for carry and no carry respectively). However, the correlation was larger in the carry condition (see Fig. 1).

### Reaction time (RT)

**Figure 2:**
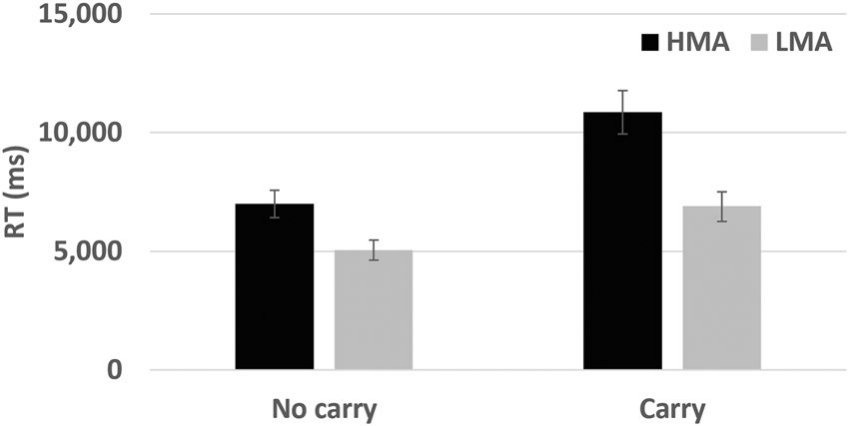
HMA participants showed longer (reaction times) RT in math problem solution; this tendency was stronger in complex carry operations than simple non-carry operations. RTs to solve mathematical operations by problem type (carry or no carry) and group (HMA or LMA).

A two-way analysis of covariance (ANCOVA) was performed on the RTs with problem type (carry or no carry) as the within subject factor and group (HMA or LMA) as the between subject factor. Tower of Hanoi and accuracy in non-symbolic comparison were the covariates. The effect of group reached significance, *F*(1, 44) = 10.26, partial *η*^2^ = 0.19, *p* < 0.01, the HMA participants had longer RT’s compared to the LMA group (*M* = 8,916 ms *SD* = 3,576 and *M* = 5,964 ms *SD* = 2,601 for the HMA and LMA groups respectively). Importantly, there was a significant group and problem type interaction, *F*(1, 44) = 7.08, partial *η*^2^ = 0.15, *p* < 0.01. The differences between the time to respond to carry and no-carry problems was much larger in the HMA group than the LMA group (*M* = 3,868 *SD* = 3,211 and *M* = 1,828 *SD* = 1,527, for the HMA and LMA groups respectively, *t*(46) = 2.85, *p* < 0.01. There were no other significant main effects or interactions (See Fig. 2).

### Proportion of use of the ten common strategies

A three-way analysis of covariance (ANCOVA) was performed on the proportion of use of the ten common strategies with problem type (no carry vs. carry) and strategy (rounding the first operand down, rounding both operands down, columnar retrieval, rounding the first operand up, rounding the second operand up, unit addition, others, vertical imagery, decomposition of the units, decomposition of the decade) as within participant factors and group (HMA or LMA) as between participant factors. Tower of Hanoi and accuracy in non-symbolic comparison served as the covariates.

The only significant main effect was strategy use, *F*(9, 396) = 2.53, partial *η*^2^ = 0.054, *p* < 0.01. Participants, across groups, used the columnar retrieval strategy significantly more frequently than the other strategies. The interaction between problem type and strategy was significant, *F*(9, 396) = 1.98, partial *η*^2^ = 0.043, *p* < 0.05. Columnar retrieval and rounding the first operand down were less frequent in the carry condition (*M* = 50%, *SD* = 36% and *M* = 0.23%, *SD* = 1%) compared to the no carry condition (*M* = 74%, *SD* = 30% and *M* = 0.8% *SD* = 2%), *t*(47) = 9.02, *p* < 0.01 and *t*(47) = 2.19, *p* < 0.05. Yet, four other strategies, rounding the two operands down, rounding the first operand up, rounding the second operand up and decomposition of the units, were used more frequently in the carry condition (*M* = 13%, *SD* = 25%, *M* = 9% *SD* = 9%, *M* = 12% *SD* = 12% and *M* = 3% *SD* = 8% respectively) compared to the non-carry condition (*M* = 4% *SD* = 15%, *M* = 1% *SD* = 3%, *M* = 1% *SD* = 3%, and *M* = 0.2% *SD* = 1% respectively) *t*(47) = −3, *p* < 0.01, *t*(47) = −5.73, *p* < 0.01, *t*(47) = −7.4, *p* < 0.01 and *t*(47) = −2.3, *p* < 0.05.

**Figure 3:**
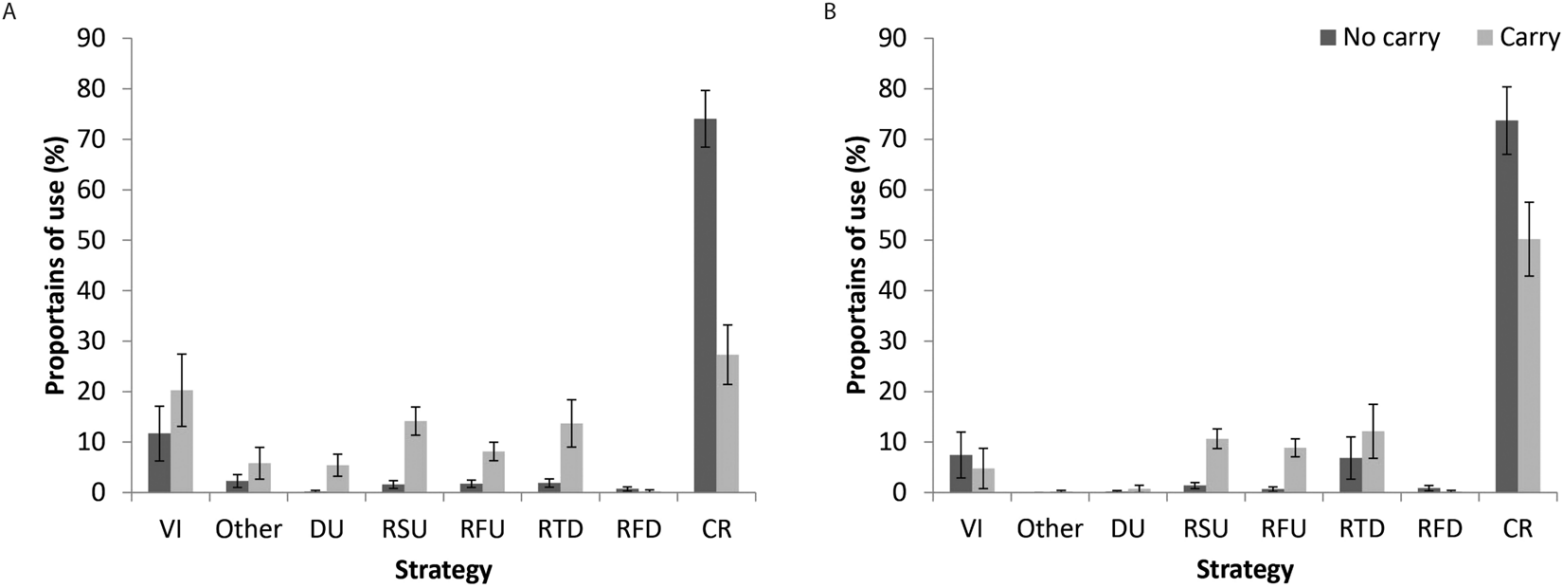
Proportion of use by strategy, problem type and group; HMA- A and LMA- B. In the easy no carry condition, most of the participants, regardless of group, used the common columnar retrieval strategy. However, in the harder carry condition, both of the groups presented a reduction in the proportion of use of the common strategy. The reduction was larger in the HMA participants compared to the LMA participants. Moreover, the strategies that were chosen instead of the common strategy in the carry condition were different between the groups: HMA tended to use decomposition of the units, vertical imagery and other strategies. VI = vertical imagery. Du- decomposition of the units, RSU- rounding second operand up. RFU- rounding first operand up. RTD- rounding two operands down. RFD- rounding first operands down. CR- columnar retrieval.

Importantly, the interaction between problem type and strategy was modulated by group, *F*(9, 396) = 5.41, partial *η*^2^ = 0.11, *p* < 0.01. In the non-carry condition participants with high and low MA used the columnar retrieval strategy very frequently (*M* = 74%, *SD* = 27% and *M* = 74%, *SD* = 33%, for HMA and LMA respectively). While both groups employed this strategy significantly less in the carry condition (*M* = 27%, *SD* = 28% and *M* = 50%, *SD* = 37% for HMA and LMA respectively), this difference was significantly larger in the HMA group compared to the LMA group, *t*(46) = −2.28, *p* < 0.05. Moreover, in the non-carry condition participants with high and low MA used the other strategies in similar frequencies (but the other strategies were more frequent in the HMA group compared to the LMA group *t*(46) = 1.85, *p* = 0.07). However, in the carry condition, the HMA group used decomposition of units more frequently, *t*(46) = 2.1, *p* < 0.05, and tended to use vertical imagery more frequently, *t*(46) = 1.92, *p* = 0.06, and other strategies, *t*(46) = 1.83, *p* = 0.07, than the LMA group (vertical imagery: *M* = 20% *SD* = 7% compared to *M* = 5% *SD* = 4% for the other frequencies see Fig. 3) (see Fig. 3).

**Figure 4:**
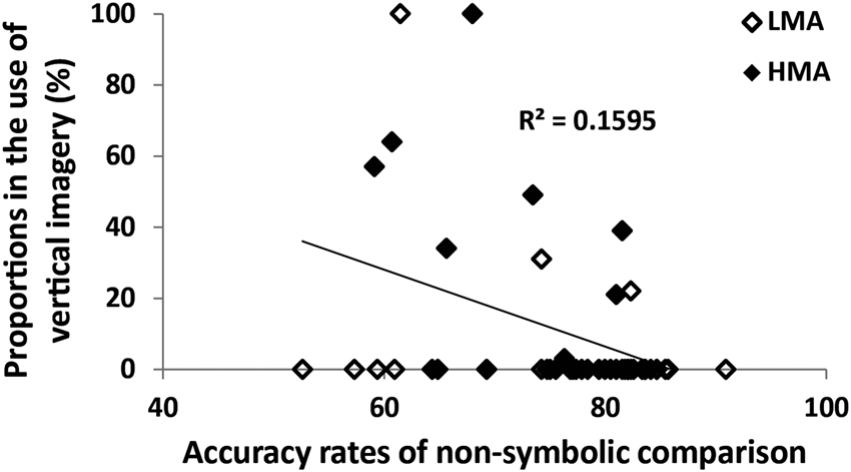
Proportion of use of the strategy vertical imagery by non-symbolic comparison abilities. Individual quantity discrimination abilities were negatively associated to the percent of usage of the strategy vertical imagery.

Interestingly, accuracy in the non-symbolic comparison task (accuracy rates of the ANS task) and strategy interacted *F*(9, 396) = 2.7, *η*^2^ = 0.39, *p* < 0.01. Hence, we tested the correlation between accuracy of the non-symbolic comparison and strategy use for each of the strategies. Non-symbolic comparison accuracy negatively associated to vertical imagery *r*(48) = −0.40, *p* < 0.01 (see Fig. 4). There were no additional significant correlations with any of the other strategies. There was no main effect or interactions with Tower of Hanoi score.

### Number of strategies used

A two-way analysis of covariance (ANCOVA) was performed on the number of used strategies with problem type (carry or no carry) as the within subject factor and group (HMA or LMA) as the between subject factor. Tower of Hanoi and accuracy in non-symbolic comparison served as the covariates. None of the effects or interactions reached significance. The only marginally significant effect was group, *F*(1, 44) = 2.86, partial *η*^2^ = 0.06, *p* = 0.098. The HMA group tended to use more strategies than LMA group (*M* = 3.26, *SD* = 1.54, *M* = 2.68, *SD* = 1.44 for HMA and LMA respectively).

## Discussion

The current study explored arithmetical strategy selection among young adults with HMA. The complex addition problems included two levels of difficulty: 1) problems with carryover, which had higher WM demands, and, 2) non-carry problems, which had lower WM demands. There is an ongoing debate on the origin of MA, and the situational influences of MA in the case of high anxiety. While some studies found that MA originates from a weakness in basic numerical processing mediated by impaired spatial abilities^5,6^, others have suggested that MA results in situated impairment in executive functions such as inhibition and shifting^33,34^. Examination of strategy selection allowed us to compare these theories among HMA participants. If impaired numerical abilities and spatial weakness characterizes MA, then participants with HMA would have difficulty selecting efficient strategies and would employ atypical strategies instead. While, executive function impairment would result in smaller variability in the number of strategies selected among HMA participants.

We found that HMA participants took longer to solve the arithmetic problems across problem type, but the difference between the groups response times was greater for carryover problems. In addition, HMA participants employed unusual strategies more often than LMA participants. Some of these strategies involved rigid spatial representation, such as vertical imagery. Similar to the RT results, we found larger group differences in strategy selection for carryover problems compared to non-carry problems. Specifically, both groups used the common strategy less often for carryover problems compared to non-carry problems; however, this difference was much larger among the HMA participants compared to the LMA participants. Instead of using the common strategy, the HMA participants used atypical strategies such as vertical imagery. In addition, vertical imagery negatively associated with innate quantity discrimination ability.

The present study found that HMA individuals were much slower in solving carry operations compared to LMA individuals, group differences were reduced in the non-carry condition. It has been widely documented that the effect of MA on mathematical task performance is modulated by task demand^2–4^. For example, a previous study found that performance on simple addition and multiplication operations (e.g., 2 + 4 or 6 × 4) was minimally impacted by MA^2–4^, while it had a greater impact on complex two-column addition problems (e.g., 21 + 18), and more so for carryover problems. The main difference between these kinds of arithmetic problems is that the solution for smaller, one-digit operations are typically retrieved from long term memory and therefore require minimal WM resources; while two-column addition problems, and more specifically carry problems, are calculated and require greater WM resources^2–4^.

One explanation for difficulty in mathematics tasks with high WM demands among HMA participants is that their WM resources are tapped out due to verbal ruminations, and therefore, have reduced WM resources available for the task at hand^2–4^. The present study suggests an alternative explanation that HMA participants select unusual, non-adaptive strategies for mathematical tasks with higher WM demands; as was seen in the difference between strategies selected for carryover versus non-carry problems.

The current study expands upon previous studies that have found that HMA participants have a particular weakness in carryover problems^2–4^, because it is the first to explore the strategy selection and cognitive processes underlying this weakness. Hodzik and Lemaire^24^ compared younger and older adults’ strategy selection for arithmetic problems, and found that high central executive abilities among the younger adults resulted in larger strategic variations and selection of the best available strategy. This finding sheds light on the relationship between MA and strategy selection due to reduced central executive abilities during situations of high anxiety^8^. Accordingly, HMA participants should act as older adults, and display lower strategic variations, and impaired selection of the best strategy. In line with this view, a study that examined strategy selection among children found that HMA children used direct retrieval (the best strategy) less often than LMA children during the solution of simple arithmetical operations^27^. However, in the current study, HMA adults displayed a tendency towards higher strategic variability compared to LMA. Please note that for adults direct retrieval is not the most sophisticated strategy for solving complex problems, and the best strategy relates to specific characteristics of each problem^16,18,24,26,28^. Dowker^23^ compared college students based on their major and found that English majors were less accurate in a computational estimation task compared to psychology students. In addition, English majors showed larger strategic variations and reduced ability to select the best strategy compared to psychology students.

The results of the present study were in line with Dowker’s^23^ findings. HMA participants in the current study showed a tendency towards larger strategic variations compared to LMA participants, especially in the harder carry condition. In addition, in line with the previous results, HMA participants tended to use less sophisticated strategies compared to LMA participants. Specifically, HMA participants tended to use “other” strategies and vertical imagery more often. The HMA group used vertical imagery in 20% of the carry trials compared to only 5% in the LMA group. In the vertical imagery strategy, participants spatially imagine writing a vertical representation of the operation. This strategy is based on spatial representation in a very primitive form.

Mature representation of number originates from preverbal spatial representation of quantity in the form of the mental number line^12,13^. The connection between quantities and space emerges during infancy^35^. Hence, reduced spatial abilities could result in a weak mental number line and later weakness in the mature representation of numbers, which can contribute to MA. Accordingly, a new line of studies found a connection between MA and reduced spatial abilities^5,6,10,15^. For example, we found that spatial WM span negatively associated to MA^6^. Moreover, Maloney *et al*.^10^ found that weakness in spatial abilities among women was the origin of higher rates of MA in women compared to men.

Interestingly, Georges *et al*.^5^ examined numerical distance effect and SNARC effects as indicators of the spatial representation of the number line in HMA participants compared to LMA participants. A steeper distance effect and a stronger SNARC effect were observed in HMA participants. The authors concluded that the stronger associations between number and space in HMA participants resulted in a reliance on concrete spatial representation preventing an understanding of abstract and complex mathematical concepts. In line with this view Cipora *et al*.^36^ found that professional mathematicians, unlike matched controls, did not reveal a SNARC effect. They suggested that professional mathematicians have more abstract or flexible spatial representations of numerical information than matched controls. In line with Georges *et al*.^5^ conclusions and supported by Cipora *et al*.’s^36^ finding, here we found a reliance on concrete spatial representation in some of the strategies that were reported in HMA participants, such as vertical imagery. This reliance may prevent HMA participants from selecting the best strategy and influence how efficiently they solve mathematical operations.

In line with the view that selection of unusual strategies characterizes MA, we found an association between basic weakness in quantity representation abilities and the tendency of using unusual strategies. For complex problem solving among adults, the best, most sophisticated strategy relates to the problem characteristics and retrieval is uncommon^16,18,24,26,28^. In order to select the best strategy, one approximates the quantity of each operand and then selects an appropriate strategy for those approximate quantities. For example, in order to solve 69 + 71, the best strategy would be rounding up the units of 69 and rounding down the units of 71. This is the best strategy for the problem due to the operands proximity to decade numbers. However, high MA individual tended to select unusual strategies regardless of problem characteristics.

In line with this view, we found in a previous study, a relationship between strategy selection and quantity discrimination^26^: better quantity discrimination negatively associated to the percent use of the common strategy (columnar retrieval) in the carry condition. That previous study found for the non-carry condition, regardless of quantity discrimination ability, most of the participants used columnar retrieval. However, in the harder carry condition, a reduction was found in the percent use of the common strategy, the reduction was larger in participants with high quantity discrimination ability than in participants with low quantity discrimination ability. A similar reduction (i.e., in the percent use of the common strategy) was found in the present study; this tendency was larger in the HMA participants compared to the LMA participants. However, the main difference between the previous study and the tendency of the HMA participants in the current study is the alternative strategy that was chosen instead of the common strategy. In the present study, a large percent of the HMA participants used non-adaptive strategies, such as vertical imagery and unusual strategies, in the carry condition. However, in Ashkenazi *et al*.^26^ a large percentage of the participants in the carry condition chose more adaptive strategies such as rounding the second operand up. In line with this view, in the present study, better quantity discrimination ability negatively associated to the percent use of the unusual strategy (vertical imagery). These results emphasize that strategic variations alone are not indicative of superior math abilities^23^. Future studies should look at the sophistication and nature of strategy selection, as well as the strategic variations in order to examine math capabilities. Spatial weakness is one possible explanation for the unusual strategies that selected in the HMA group. Other explanations can relate more directly to numerical difficulties or less practice solving complex addition problems.

In addition, low math performance can be the origin of MA, which can then result in a further drop in math performance^9,37^. Hence, one cannot fully rule out the explanation that HMA participants in the current study selected unusual non-adaptive strategies due to low math abilities. Moreover, future studies should further explore the direct relationships between the strategies frequently selected by high MA individuals (such as vertical imagery) and weakness in numerical- space associations in MA. The findings of the current study suggests weak numerical space associations among MA participants based on the unusual strategies selected for the carryover problems, but did not directly relate that tendency to spatial weaknesses.

## Conclusion

Over the last two decades it has been reported that MA impairs performance specifically in mathematical operations that involve high WM demands, such as carry problems^2–4^. Here, for the first time we uncovered the cognitive mechanism underlying the deficits in operations that require carryover by examining strategy selection. We found that the HMA participants used primitive, non-adaptive strategies grounded in space. The use of these unusual strategies was mostly observed in the harder operations that required carryover.

The innate representation of quantity found in infancy is related to space^35^; however, over the course of development and formal education, more mature symbolic numerical representation is built upon this primitive representation and creates more abstract numerical representations. Spatial weaknesses among HMA participants^5,6,10,15^ can prevent the mental construction of abstract numerical representation. Thus, they continue to use primitive numerical representations, based on spatial representations, that are not adaptive and can influence performance in operations with high WM demands.

## Material and Methods

### Participants

Forty-eight students from the Hebrew University of Jerusalem, 42 women and 6 men, participated in the experiment. They were between the ages of 18 and 30-years-old (M = 23.45 S.D = 2.38). Four of the participants were left-handed and the rest were right-handed. None of the participants reported diagnosis of ADHD or learning disabilities and all had normal or corrected-to-normal visual acuity. They were compensated with course credits or 30 NIS (approximately $8.60).

All the methods of the study were performed in accordance with the relevant guidelines and regulations. The study was approved by the local Ethics Committee of the Seymour Fox School of Education at the Hebrew University of Jerusalem. Written consent was obtained according to the Declaration of Helsinki. The participants provided written informed consent.

The participants were divided to two groups according to their scores in The Mathematics Anxiety Rating Scale - Shortened (MARS-S). Participants with scores lower than 75 were included in the LMA group. Participants with scores higher or equal to 81 were included in the HMA group. Participants with a score between 76 to 80 were not included in the present study.

Two of the participants were excluded due to long RT’s (more than 3 SD from the average), another participant was eliminated due to accuracy rates less than 50% in each of the conditions.

### Apparatus

The experimental task (complex calculation), was controlled by a Genuine-Intel compatible PC 1.73 GHz using E-prime experimental software, 2.1 version (Schneider, Eschman, & Zuccolotto, 2002). The Tower of Hanoi was controlled by PEBL (Mueller, 2012). The ANS task was controlled by the Panamath software^38^. Instructions and stimuli were presented on a 14″ monitor. The computer monitor was located approximately 50 cm in front of the participant.

### MARS-S

The Mathematics Anxiety Rating Scale (MARS) has been widely used since 1972, the MARS-S is a 30 item self-rating scale to assess MA^39^, a short version of the MARS. Participants are asked to indicate on a 5-point Likert scale from 1 (low anxiety) to 5 (high anxiety) how anxious they feel in various math-related situations. Adequate internal consistency (Cronbach’s α = 0.96), test-retest reliability (r = 0.9) and construct validity have been reported for this instrument. It was translated into Hebrew by our lab.

### Complex calculations: main task

Each participant solved 48 complex addition problems. A typical trial began with a fixation mark presented in the center of the computer screen for 300 ms, which was followed by a blank screen for 500 ms. Then the problem appeared in the form of a + b = , the participant had to orally report the answer. The problem was presented until the participant responded. Afterwards, the experimenter recorded the participants’ response. Then a screen with the question “How did you solve the problem?” appeared. The question appeared until the participant answered the question. The experimenter recorded participants’ response. The trial was finished with a blank screen for 1500 ms.

The problems in the task were composed of two two-digit numbers with a mean sum of 135.63 (S.D. = 17.89, range = 104–169). To test problem difficulty effects, problems were categorized as either “carry problems” or “non-carry problems” on the basis of the presence/absence of a carryover in the unit’s digit; all the problems required carryover in the hundreds digit (e.g. 64 + 66 = 130). Following previous findings in arithmetic (see^26^), problems were selected with several constraints: (a) half the problems had their larger operand on the left position (e.g., 68 + 37); (b) none of the operands had the tens or units digits equal to 0; (c) none of the operands had unit digits equal to 5; (d) none of the pairs of operands had the same ten and unit digits (e.g., 64 + 68, 64 + 54), the same ten and unit digits (e.g., 33 + 88), or the same operands (e.g., 71 + 71); (f) none of the problems were the reverse of another problem (i.e., if 72 + 64 was used, 64 + 72 was not). Participants were not allowed to use paper and pencil, and no feedback was provided. The experimenter could classify the reported strategy to one of the following (using the example (72 + 46)): (1) rounding the first operand down (e.g., (70 + 46) + 2), (2) rounding the second operand down (e.g., (72 + 40) + 6), (3) rounding both operands down (e.g., (70 + 40) + (2 + 6)), (4) columnar retrieval (e.g., (2 + 6) + (70 + 40)), (5) rounding the first operand up (e.g., (80 + 46) − 8), (6) rounding the second operand up (e.g., (72 + 50) − 4), (7) rounding the two operands up (e.g., (80 + 50) − 8 − 4), (8) unit addition (e.g., (78 + 40)), (9) retrieval (e.g., 118), and (10) others (none of the other strategies) (11) vertical imagery (e.g., $$+\begin{array}{l}12\\ \underline{46}\end{array}$$), (12) decomposition of the units (e.g., (2 + 6) + 2 + (70 + 40) − 2), (13) decomposition of the decade (similar to the previous one but in the decade unit).

### Tower of Hanoi

In a computerized version of this task, three rods and a number of disks of different sizes, which can slide onto any rod, were displayed on the screen. The participant was instructed to move all the discs from a start position to the end position (task from the PEBL^40^). The dependent variable was the average difference between the shortest number of steps available in a specific trial and the actual number performed by the participant.

### ANS task

Two sets of overlapping dots, one in yellow and one in blue, appeared on the screen briefly (for 300 ms.). The participants were requested to indicate if the blue cloud or yellow cloud was larger in quantity. Ratios between sets were manipulated between 1:2, 3:4, 5:6 or 7:8. For every set, the number range was between 5 and 16 dots. To control for possible intervening variables such as total area and dot size, the trials were split in half: half of the trials were controlled for area, the total area of the two sets were identical and the remaining trials were controlled for size, such that the size of the dots in both sets were equal (n = 120) for the full details see^38^.

### Materials and procedure

First, participants completed the MARS-S followed by 1) the main arithmetical task, 2) Tower of Hanoi task and 3) ANS task. Participants with a score of less or equal to 75 (minimum = 32, maximum = 75, average = 60.28, S.D., 11.42) were defined as LMA (n = 25, mean age = 23.67 S.D = 1.77), participants with a score greater or equal to 81 (minimum = 81, maximum = 118, average = 104.14, S.D. = 7.78) were defined as HMA (n = 23, mean age = 23.42 S.D = 2.9). Out of the LMA participants, 19 were females and 22 of the high MA were females. Two participants in each group were left handed.
